# Retraction Note: Analysis of antiviral efficacy after switching from brand to generic entecavir in patients with treatment-naïve chronic hepatitis B

**DOI:** 10.1186/s12876-022-02483-8

**Published:** 2022-08-26

**Authors:** Po-Ke Hsu, Pei-Yuan Su, Chia-Lin Wu

**Affiliations:** 1 Division of Gastroenterology, Department of Internal Medicine, Changhua Christian Hospital, Changhua, Taiwan; 2 Institute of Medicine, Chung Shan Medical University, Taichung, Taiwan; 3 School of Medicine, Chung Shan Medical University, Taichung, Taiwan; 4 Division of Nephrology, Changhua Christian Hospital, Changhua, Taiwan; 5 School of Medicine, National Chung Hsing University, Taichung, Taiwan

## Retraction to: BMC Gastroenterology (2022) 22:228 10.1186/s12876-022-02317-7

The authors have retracted this article as it has been published previously [1]. All authors agree with this retraction.

## References

[1] Su P-Y, Yen H-H, Huang S-P, Hsu P-K, Chen Y-Y, Hsu Y-C (2021). Switching from branded to generic entecavir in patients with treatment-naïve chronic hepatitis B: experience of a single tertiary medical center. Changhua J Med..

